# Multi-Sensor and Decision-Level Fusion-Based Structural Damage Detection Using a One-Dimensional Convolutional Neural Network

**DOI:** 10.3390/s21123950

**Published:** 2021-06-08

**Authors:** Shuai Teng, Gongfa Chen, Zongchao Liu, Li Cheng, Xiaoli Sun

**Affiliations:** 1 School of Civil and Transportation Engineering, Guangdong University of Technology, Guangzhou 510006, China; 1112009002@mail2.gdut.edu.cn (S.T.); 1111709005@mail2.gdut.edu.cn (Z.L.); s13640223065@163.com (X.S.); 2 Department of Mechanical Engineering, The Hong Kong Polytechnic University, Hong Kong 999077, China; li.cheng@polyu.edu.hk; 3 Guangzhou Municipal Engineering Testing Co., Ltd., Guangzhou 510520, China

**Keywords:** structural damage detection, decision-level fusion, 1-D convolutional neural network, vibration experiments, acceleration signals, bridge model

## Abstract

This paper presents a novel approach to substantially improve the detection accuracy of structural damage via a one-dimensional convolutional neural network (1-D CNN) and a decision-level fusion strategy. As structural damage usually induces changes in the dynamic responses of a structure, a CNN can effectively extract structural damage information from the vibration signals and classify them into the corresponding damage categories. However, it is difficult to build a large-scale sensor system in practical engineering; the collected vibration signals are usually non-synchronous and contain incomplete structure information, resulting in some evident errors in the decision stage of the CNN. In this study, the acceleration signals of multiple acquisition points were obtained, and the signals of each acquisition point were used to train a 1-D CNN, and their performances were evaluated by using the corresponding testing samples. Subsequently, the prediction results of all CNNs were fused (decision-level fusion) to obtain the integrated detection results. This method was validated using both numerical and experimental models and compared with a control experiment (data-level fusion) in which all the acceleration signals were used to train a CNN. The results confirmed that: by fusing the prediction results of multiple CNN models, the detection accuracy was significantly improved; for the numerical and experimental models, the detection accuracy was 10% and 16–30%, respectively, higher than that of the control experiment. It was demonstrated that: training a CNN using the acceleration signals of each acquisition point and making its own decision (the CNN output) and then fusing these decisions could effectively improve the accuracy of damage detection of the CNN.

## 1. Introduction

Structural damage detection (SDD) is one of the most relevant topics in structural health monitoring (SHM). Timely SDD is helpful for finding the potential defects of a structure and preventing its sudden collapse. The early detection methods are mainly on-site inspections, which are labor-intensive, time-consuming, and only effective for visible surface defects. The structural vibration contains real and complete state information of a structure [1]; therefore, some vibration-based SDD methods are proposed. For example, the SDD methods are based on modal parameters and their derivatives (namely, the parametric method), including the natural frequencies [2], mode shapes [3], modal flexibility [4], mode curvature [5], and modal strain energy [6,7]. The non-parametric method establishes the SDD indicators directly from the real-time vibration signals, including acceleration [8] and displacement [9]. Among them, the real-time detection method based on eigen perturbation and a Kalman filter has been well confirmed [10]. Although these methods have significantly improved the accuracy of the SDD, they still face many challenges. The parametric methods need accurate modal parameter identification, which may be compromised under the influence of many factors (measurement and/or analysis errors). Furthermore, a single modal-based indicator cannot cover all damage scenarios (e.g., the natural frequencies can only detect the existence of damage, but cannot determine the damage location) [2]; meanwhile, the non-parametric methods require large-scale data analysis, which is affected by the knowledge level of analysts, and the accuracy and efficiency of damage detection are questionable. Even the popular Kalman filter method also needs both accurate structural modeling and external excitation, which will limit its application in real engineering [10]. Therefore, an automatic and efficient data processing tool to integrate/fuse multiple information sources is urgently needed.

Machine learning (ML) methods provide a new way to solve the above difficulties. The ML enables a system to automatically learn from its experience and predict the corresponding scenario according to the learned knowledge. ML algorithms have been widely used in vibration-based SDD. Classical ML algorithms include the support vector machine (SVM) [11] and artificial neural network (ANN) [12], which have achieved encouraging results. In particular, the backpropagation (BP) neural network has been widely applied to the parametric and non-parametric SDD methods, for example, damage detection of a truss [13], a steel frame [14], and a bridge model [15], and its effectiveness was also confirmed on a real steel frame [16]. However, all the above methods need to extract a set of fixed features, e.g., the modal parameters and/or wavelet transform coefficients [17], principal component analysis (PCA) [18], and wavelet decomposition (WD) [11]. Furthermore, the fully connected neural network (i.e., BP neural network) is prone to over-fitting and is computationally expensive, which will sacrifice the effectiveness of the method in large-scale SDD tasks.

As a deep learning algorithm, a convolutional neural network (CNN) provides a novel method for the SDD due to its excellent feature extraction ability. Meanwhile, a CNN has powerful computing performance and is able to prevent over-fitting due to its weight sharing (in the convolution process) and sparse connection (in the pooling process); it has unprecedented potential in the field of SDD. Zhong et al. [19] demonstrated that a CNN can extract damage information from the mode shapes; Lin et al. [20] also showed that a CNN can extract damage information directly from the acceleration signals, and Teng et al. [21] illustrated a CNN feature extraction process in structural surface defect detection. The effectiveness of a 2-D CNN was demonstrated using numerical [22] and experimental [23] models of a benchmark structure by joining the data of 14 accelerometers. As an alternative, a 1-D CNN has attracted attention in electrocardiogram (ECG) detection, engine detection [24], and voltage/current detection of electronic equipment [25]. These studies confirmed the excellent performance of a 1-D CNN in damage detection. In the field of civil engineering, a 1-D CNN was used [26] to detect damage in a laboratory frame, where its effectiveness was validated on the collected acceleration signals using a wireless sensor network (WSN) [27]. Subsequently, the SDD method based on the vibration and 1-D CNN was also used to detect the mass changes of the real bridges [28]. Although the CNN-based SDD methods achieved encouraging results, for practical engineering, especially for the long-span bridges, it is difficult to collect the complete bridge vibration information and arrange sufficient signal acquisition points. Therefore, although a CNN has a strong signal processing capability, the damage detection is affected by the non-synchronization and incompleteness of the vibration signals and the interference between multiple sensors. In order to obtain more complete damage information, a new data analysis strategy is necessary.

The strategy of data fusion provides a state-of-the-art SDD method. By fusing multi-channel/multi-scale information, the data fusion technology can provide complete and detailed object information. In medical engineering, computed tomography and magnetic resonance (CT-MR) image fusion can obtain a more accurate lesion location [29]; in remote sensing image processing, image fusion technology can improve image resolution [30]. In the field of SHM, the time domain and frequency domain images of the bridge vibration were fused to detect abnormal signals [31], and the accuracy of damage detection was improved by fusing the modal strain energy (MSE) of multi-modes [32] and the MSE with dynamic response [33], and the Dempster–Shafer (D–S) evidence theory and multi-sensor-signals-based SDD method was also implemented [34]. The damage indicators based on modal parameters and their derivatives need accurate modal identification from the original vibration signal and the accuracy is compromised by the accidental error of measurement and/or analysis. The popular Kalman filters can effectively eliminate the interference of noise [35]; however, the structural parameter identification method based on eigen perturbation and a Kalman filter still faces many challenges: (1) it can only be used to identify time-invariant structural parameters [36]; (2) for sub-component (location) damage detection of a structure, the accuracy and robustness need to be further improved [37]; (3) it cannot be applied to a non-Gaussian parameter system [38]; (4) there is a certain time delay [39]; (5) low sampling frequency will affect the stability of the filter [40]. These often lead to significant implementation difficulties. The vibration signals contain the complete structural state information [41]; thus, it is of great potential to use the vibration signals as structural damage indicators. The information of a single sensor has a certain ability to detect the structural damage state [42]; however, the influence of the sensor location on damage detection results is not clear, and the complementarity of multiple sensors is also a topic worthy of further study. The existing methods fuse the original data of multiple sensors as the input of a CNN (namely, data-level fusion); however, the collected signals may be unsynchronized and incomplete, and the signals of multiple sensors may have interference.

In order to further improve the accuracy of damage detection, one solution was to synthesize the information of multiple sensors and avoid mutual interference. In this study, a novel decision-level fusion strategy was applied to the SDD. That is, each acquisition point (accelerometer) was regarded as an independent observation unit. Each accelerometer signal was used to train a 1-D CNN, and the prediction results of the multiple CNN models were integrated to finally predict the structural damage state (decision-level fusion). This work was carried out on a numerical model and two experimental models; meanwhile, a control experiment (data-level fusion) was designed to highlight the advantages of the proposed method.

## 2. Materials and Methods

**Figure 1:**
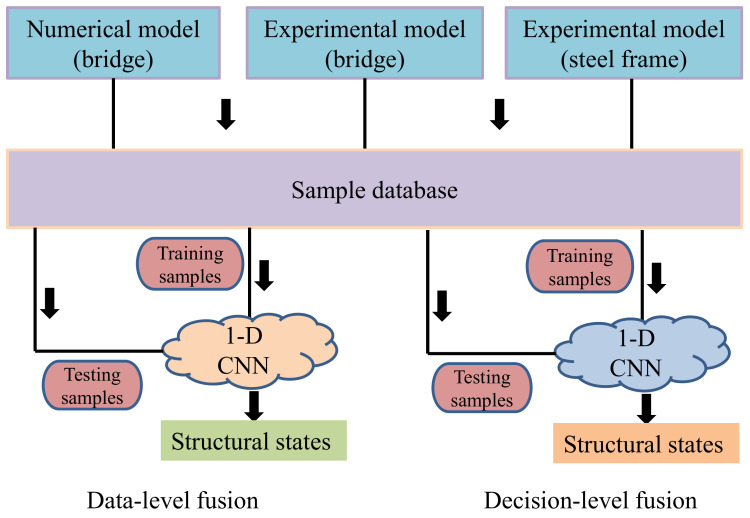
The implementation strategies of the SDD using the 1-D CNN.

In this study, 3 cases of damage detection were carried out, including numerical and experimental models of a bridge structure and a large-span steel frame model. The detailed implementation strategies were as follows (Figure 1).

### 2.1. Numerical and Experimental Models

**Figure 2:**
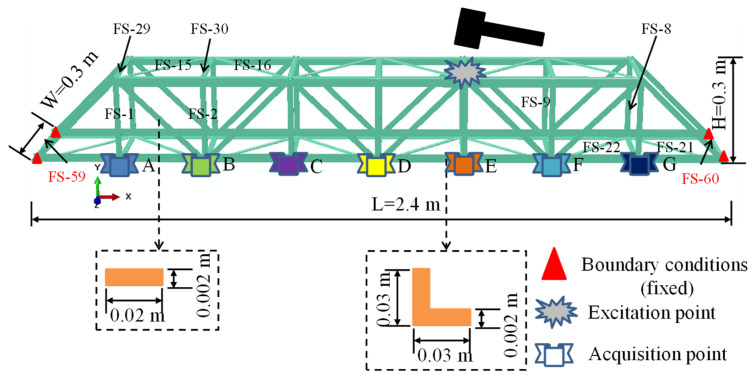
The bridge model with 60 flat steel bars.

**Figure 3:**
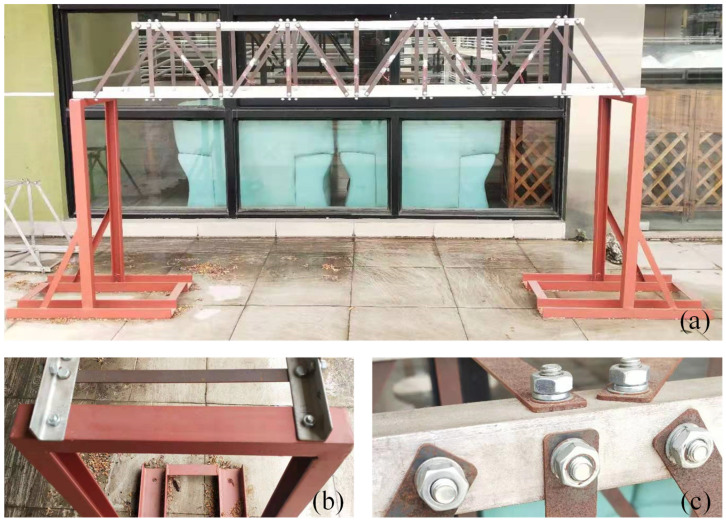
The bridge model with 60 flat steel bars. (**a**) Model layout; (**b**) boundary condition; (**c**) connection type.

The numerical model (Figure 2) of the bridge structure (Figure 3a) with a length of 2.40 m, a width of 0.30 m, and a height of 0.30 m was created in ABAQUS (SIMULIA Inc, Providence, RI, USA); it included 60 flat steel bars. Each flat steel bar had a rectangular cross-section (0.02 × 0.002). The elastic modulus, Poisson’s ratio, mass density, and modal damping ratio of the flat steel were 210 GPa, 0.3, 7800 kg/m^3^, and 0.003, respectively, for the bridge model. All the flat steel bars were meshed with beam elements (B31 type). The 60 flat steel bars were named FS-1, FS-2, …, FS-60, respectively; among them, FS-59 and FS-60 were not used as the investigated objects because their 2 ends were fixed, and their damage had no effect on the vibration signal, which was shown in relevant studies [33].

**Figure 4:**
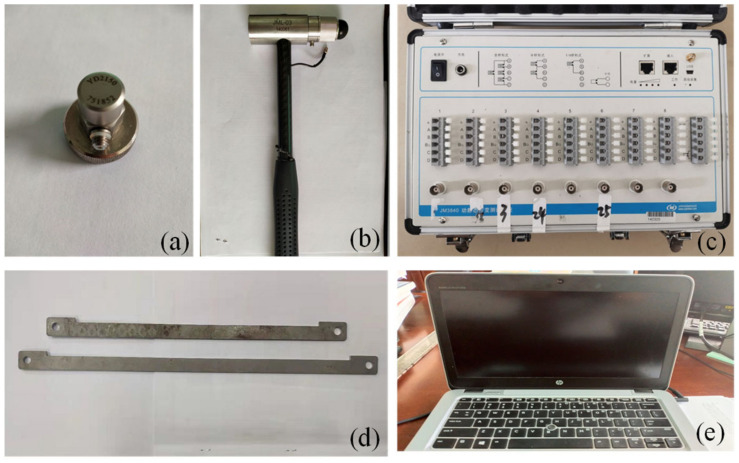
Experimental facilities: (**a**) accelerometers; (**b**) hammer; (**c**) JM3840 dynamic data acquisition instrument; (**d**) damaged flat steel bars; (**e**) laptop.

The bridge structure in Figure 3a was used for the vibration experiment. The flat steel bars were connected using bolts (Figure 3c). The experimental facilities (Figure 4) included: a JM3840 dynamic data acquisition instrument, 7 accelerometers, an instrumented hammer, a laptop, and some damaged flat steel bars (damage scenarios were achieved by replacing the flat steel bars in the damaged location). Figure 3b showed the boundary conditions of the experimental model (it was pinned on the supporting frame using the bolts).

**Figure 5:**
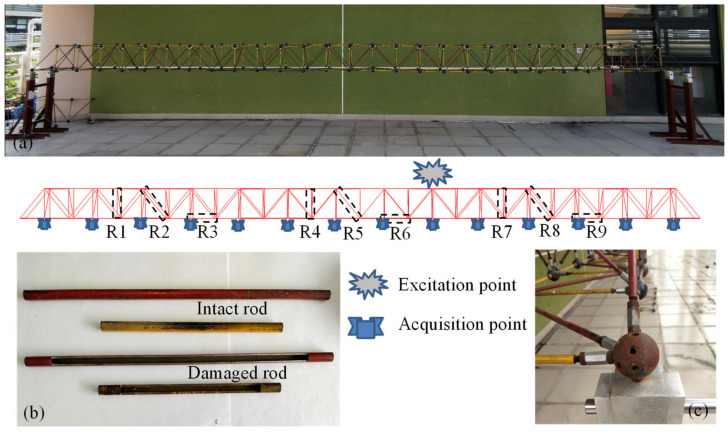
The steel frame model with 355 rods. (**a**) Model layout; (**b**) intact and damaged rods; (**c**) boundary condition.

In order to further validate its generality, the proposed method was also applied to a long-span steel frame model (Figure 5). The steel frame had a length, width, and height of 9.912 m, 0.354 m, and 0.354 m, respectively. The steel frame consisted of 355 rods; each rod had a hollow circular cross-section with an external radius of 0.005 m and thickness of 0.002 m. The 2 ends of the steel frame were pinned. Damage was introduced to 9 rods (namely, R1, R2, …, R9 in Figure 5). The response signals of 13 acquisition points (accelerometers) on the bottom chord were used as the inputs of the CNN. The excitation point was on the top chord (Figure 5).

### 2.2. 1-D Convolution Neural Network

**Figure 6:**
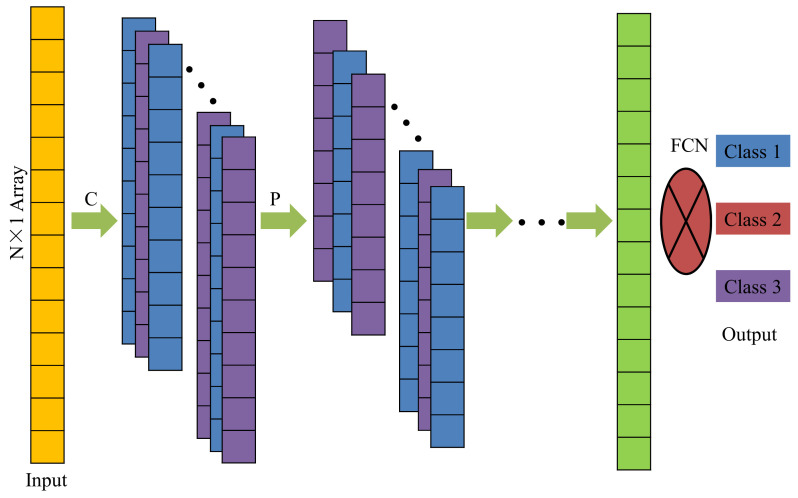
Architecture of a 1-D CNN. C: convolution layer; P: pooling layer; FCN: fully connected layer.

A standard CNN usually includes a series of convolution layers, pooling layers, activation layers, a fully connected layer, a softmax layer, and an output layer. The input data is transferred through these layers, and finally, it is mapped to the class to which the original data belongs. In particular, the input of a 1-D CNN is a 1 × N or N × 1 array. As shown in Figure 6, an N × 1 array goes through a series of convolution and pooling layers, and finally, finds the class (class 1, class 2, or class 3) of the array in the output layer.

**Figure 7:**
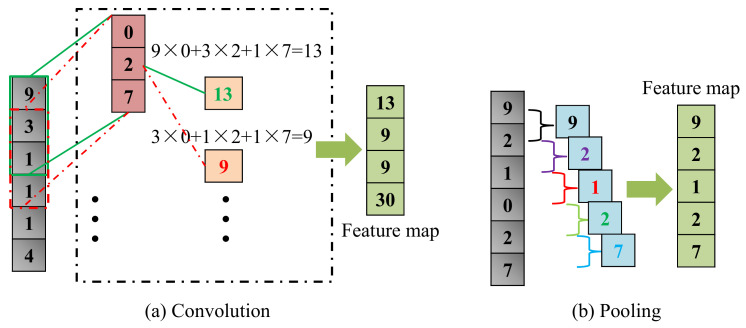
Convolution and pooling operations. (**a**) Convolution; (**b**) pooling.

The convolution process (Figure 7a) involves multiplying each element in the convolution kernel with the corresponding element in a sub-region (e.g., green box or red dotted box) of the input data of the convolution layer and summing up the products to obtain an element in the feature map. Each time, the sub-region moves down 1 step and the process is repeated until all elements of the input data are involved; in the end, the convolution operation will form a new array (i.e., the feature map).

The pooling operation is a down-sampling technique that greatly improves the CNN computational speed and effectively prevents over-fitting. There are usually 2 different pooling methods, namely, max pooling and mean pooling. Max pooling was utilized in this study as it is better than mean pooling [43]. Figure 7b demonstrates that max pooling picks up the maximum value of a sub-matrix (2 × 1) to form an element of the feature map.

The activation layers, softmax layer, and fully connected layer are similar to a general 2-D CNN, which was described in a relevant reference [33]. The responses of a structure to the excitation were different under different damage scenarios. In this study, the vibration signals (acceleration) of multiple acquisition points (accelerometers) of the bridge model or steel frame model were taken as the input of the network, and the damage state of the structure was taken as the output (e.g., different damage locations were labeled as different scenarios). In the process of network training, the 1-D CNN used the convolution and pooling layers to process the acceleration signals layer by layer to extract the damage information, which was classified into different damage scenarios in the fully connected layer.

### 2.3. Structural Damage Detection

First, the vibration signals of various structural scenarios (one intact structure and 58 damaged structures (the damage locations were FS-1, FS-2, …, FS-58, respectively) were obtained by using the numerical model described in Section 2.1, where the parametric analysis codes based on ABAQUS and PYTHON were reported in a relevant reference [32]. The damage of the flat steel bar was simulated using the change of its elastic modulus. It was assumed that the damage level of the flat steel bar was proportional to the reduction of its elastic modulus. In this study, the elastic modulus of the flat steel bar at the damage location was reduced by 60%. Two consecutive impulse excitations (800 N and 1000 N) were applied to the structure at the excitation point, and then the acceleration signals of 400 sampling points (sampling time of 4 s with an increment of 0.01 s) of each impulse excitation were collected. The CNN samples were created as follows.

**Figure 8:**
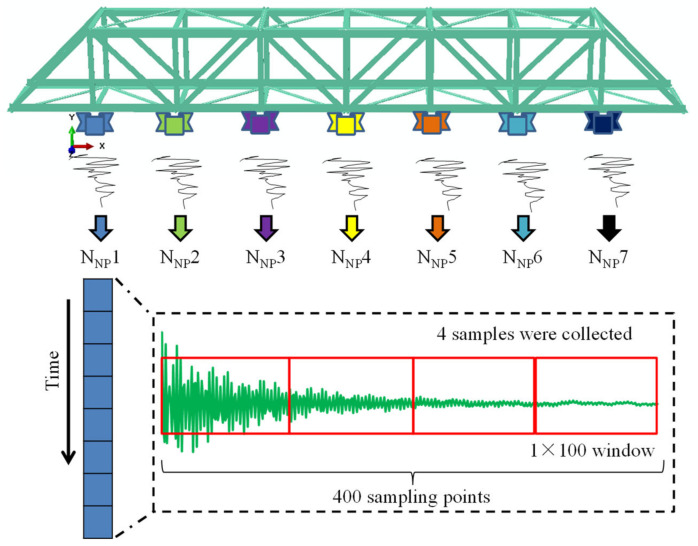
The sample acquisition of the CNNs.

As shown in Figure 8, the vibration signal (1 × 400 array) generated by an excitation, with its 400 sampling points, was divided into 4 equal parts through the fixed size windows, that is, 4 samples (four 1 × 100 arrays); this operation was repeated to obtain all 472 samples (4 × 59 (1 intact structure and 58 damage locations) × 2 (2 excitations)). The samples from the 1000 N excitation were used as the training samples (236 samples), and the samples from the 800 N excitation were used as the testing samples (236 samples). The CNN input was acceleration signals, and the CNN output was labeled as state 1 (intact structure), state 2 (damage on FS-1), state 3 (damage on FS-2), and so on. The acceleration signals from each acquisition point (7 points in total) were used to train each respective CNN, that is, there were a total of 7 CNN models (namely, N_NP_1, N_NP_2, N_NP_3, …, N_NP_7, as shown in Figure 8).

**Figure 9:**
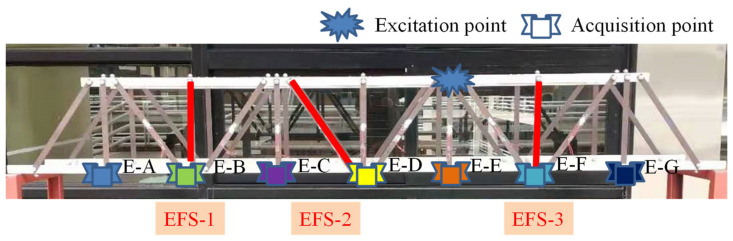
Distribution of the damage locations and accelerometers (bridge model).

Second, for the design of the damage scenarios of the experimental model (bridge model), the intact flat steel bar was replaced with a damaged flat steel bar of the experimental model, and the following 6 structural scenarios were designed: state 1 (intact structure), state 2 (damage on EFS-1), state 3 (damage on EFS-2), state 4 (damage on EFS-3), state 5 (damage on EFS-1 and EFS-2, simultaneously), and state 6 (damage on EFS-1, EFS-2, and EFS-3 simultaneously). For each damage scenario, the structure was stimulated 3 times (at the excitation point by a hammer) and the acceleration signals were collected at the corresponding locations (E-A, E-B, E-C, …, E-G in Figure 9), where the data obtained from the 1st and 2nd excitations were used as the training samples and the data from the 3rd one was used as the testing samples. According to the above sample acquisition method (Figure 8), for a CNN sample dataset, the number of training and testing samples was 48 (4 × 6 (6 structural scenarios) × 2 (2 impulse excitations)) and 24 (4 × 6 (6 structural scenarios) × 1 (1 impulse excitation)), respectively. Seven CNN models could be trained from the acceleration signals collected by the 7 accelerometers (namely N_NP_1, N_NP_2, N_NP_3, …, N_NP_7).

Third, according to the above method, a total of 10 structural scenarios were designed in the steel frame model (Figure 5), i.e., state 1 (intact structure), state 2 (damage on R1), state 3 (damage on R2), and so on. According to the above sample acquisition method (Figure 8), for the CNN sample dataset, the number of training and testing samples was 80 (4 × 10 (10 structural scenarios) × 2 (2 excitations)) and 40 (4 × 10 (4 structural scenarios) × 1 (1 excitation)), respectively. In total, 13 CNN models could be trained from the acceleration signals collected by the 13 accelerometers (namely, N_NP_1, N_NP_2, N_NP_3, …, N_NP_13).

Subsequently, a 1-D CNN was established by using the ‘Deep Learning Toolbox’ of MATLAB (MathWorks Inc., Natick, MA, USA), including 2 convolution layers, 1 pooling layer, 2 activation layers (leaky ReLU activation function), 1 fully connected layer, and 1 softmax layer. Detailed network parameters are shown in Table 1.

In this study, 7 CNNs (for the bridge model) could be obtained from 7 acquisition points (accelerometers). The testing samples were used to evaluate the performance of 7 networks, and the testing results of each network were fused (decision-level fusion) as follows.

The prediction results of the 7 networks were P1, P2, …, P7:

(1) $$P_{i}=\left[a_{i}\;b_{i}\;c_{i}\;\ldots \right]$$

where *i* = 1, 2, … 7, and *a_i_*, *b_i_*, *c_i_*, etc., represent the prediction results of the *i*th network for the first, second, and third testing samples, and so on. In this study, the decision fusion of predictions (DFP) was calculated from the predicted results of the 7 networks:

(2) $$P_{T}=\left[\begin{array}{c} P_{1}\\ \vdots \\ P_{7} \end{array}\right]=\left[\begin{array}{c} a_{1}\;b_{1}\;c_{1}\;\ldots \\ \vdots \\ a_{7}\;b_{7}\;c_{7}\;\ldots \end{array}\right]$$

(3) $$DFP=Mode\left(P_{T},2\right)$$

where Mode is a MATLAB function; Mode (***P****_T_*, 2) was used to calculate the most frequent number in each column of the ***P****_T_*. This was similar to the voting process in an election, where all the decision makers (i.e., the CNNs) vote on the decisions, and the decision with the most votes is recognized. 

**Figure 10:**
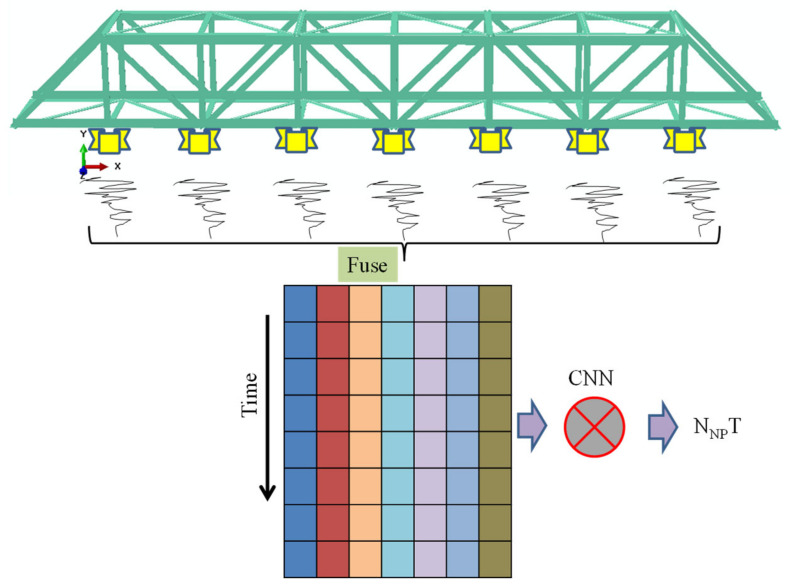
Implementation of the control experiment (data-level fusion).

In order to further prove the outstanding performance of the proposed method, a corresponding control experiment was designed: the acceleration data of all acquisition points were fused (as shown in Figure 10) using data-level fusion. Therefore, the CNN input was a 2-D array, and then a sample database was established by using the data of all structural states (it was consistent with the numerical and experimental models described in Section 2.3), and the training samples were input into the CNN model (N_NP_T) to implement the network training.

## 3. Results and Discussion

This section includes two parts: (1) damage detection results (decision-level fusion) based on the numerical model and 1-D CNN, and comparisons with that of the control experiment (data-level fusion); (2) the damage detection results of two experimental models (the bridge model and long-span steel frame model), and comparisons with that of the control experiment (data-level fusion).

### 3.1. Detection Results of the Numerical Model

**Figure A1:**
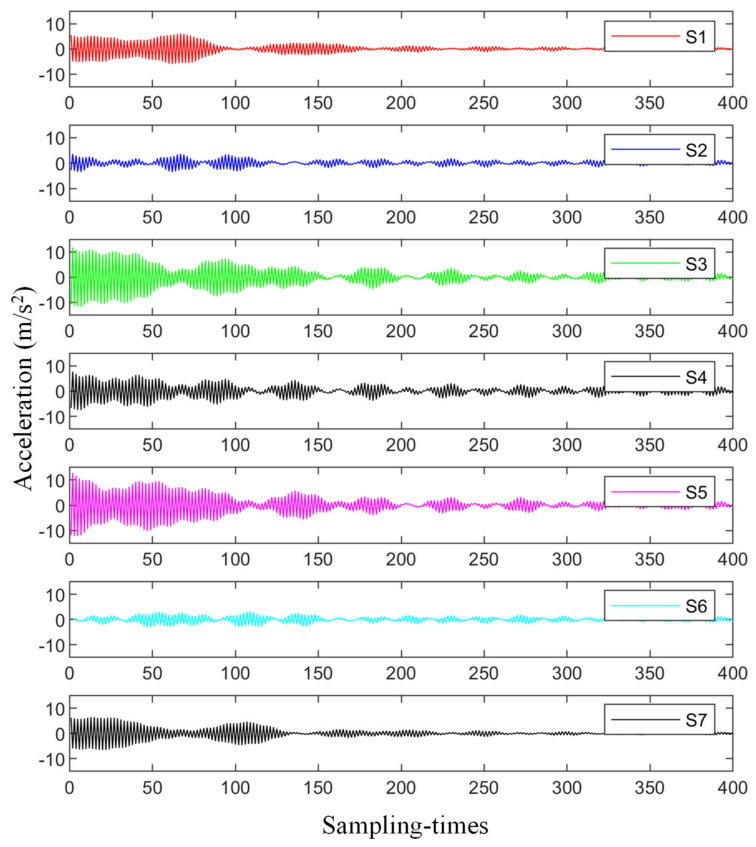
Acceleration signals from the numerical model.

First, ABAQUS was employed for the numerical simulations of the bridge model. The vibration signals of the intact structure are shown in the Appendix A (Figure A1), where S1, S2, …, S7 are the time history curves of the acquisition points (A, B, …, F, G) under the 800 N impulse force excitation, respectively; the complete vibration signals (for all structural scenarios) are shown in the Supplementary Materials. The training and testing samples of the 1-D CNN were obtained by using the method described in Section 2.3.

**Figure 11:**
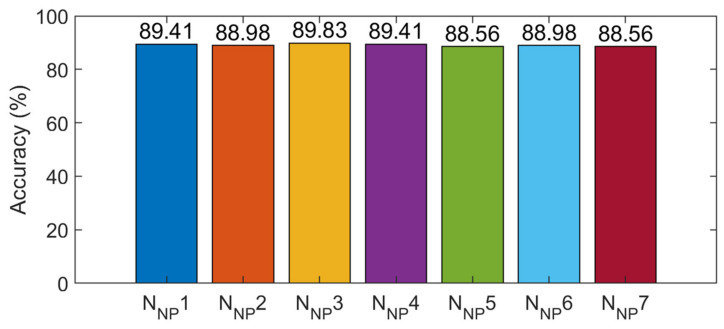
Detection results of the 7 networks.

**Figure A2:**
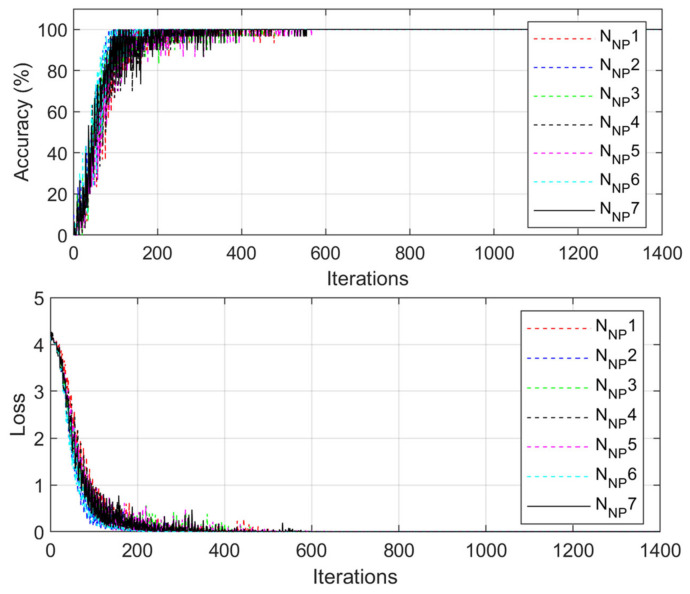
Training process and detection results. N_NP_#: network number.

The training samples of the numerical bridge model were used to train the 1-D CNN. The training processes of the seven networks are shown in the Appendix A (Figure A2). After 1400 iterations, the accuracy and loss value of the networks tended to be stable, the accuracy of the training samples was 100%, and the loss value tended to be 0. The testing samples were used to evaluate the detection performance of the 1-D CNN; Figure 11 shows the accuracy of the testing samples of the seven networks N_NP_1 to N_NP_7, ranging from 88 to 90%.

Table 2 shows some detection errors of the seven networks for the testing samples, where the mistakenly detected cases of each network were different. For example, N_NP_1, N_NP_2, and N_NP_4 had incorrect detections for structural state 1, while N_NP_3, N_NP_5, N_NP_6, and N_NP_7 had no wrong detections for state 1; N_NP_1 and N_NP_6 had incorrect detections for structural state 57, while N_NP_2, N_NP_3, N_NP_4, N_NP_5, and N_NP_7 had no wrong detections for state 57. Therefore, each CNN had different sensitivities to different damage scenarios.

**Figure 12:**
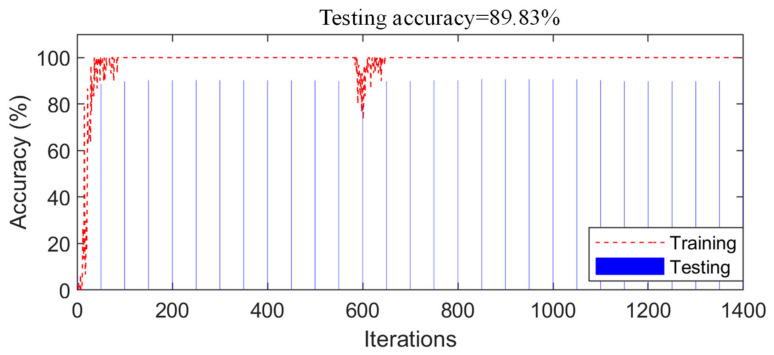
Detection results of the control experiment (numerical bridge model).

According to the proposed fusion strategy described in Section 2.3, the prediction results of all network models were fused and the results showed that the accuracy of the SDD using the decision-level fusion strategy was 100%. The training process of the control experiment (data-level fusion) is shown in Figure 12; in the stable stage of the network training, the accuracy of the training samples reached 100%. Figure 12 also shows the change in the accuracy of the testing samples for the different iterations. Finally, the accuracy of the testing samples was 89.83%; therefore, the accuracy of the proposed method was higher than that of the control experiment (data-level fusion). Furthermore, the accuracy of the decision-level fusion strategy was higher than that of any individual network before the fusion, as shown in Table 3. Hence, interestingly, any individual network (before fusion) could only achieve about 90% accuracy; the accuracy was improved by about 10% by using the proposed fusion strategy to fuse the results of multiple networks.

### 3.2. Detection Results of the Experimental Model

**Figure A3:**
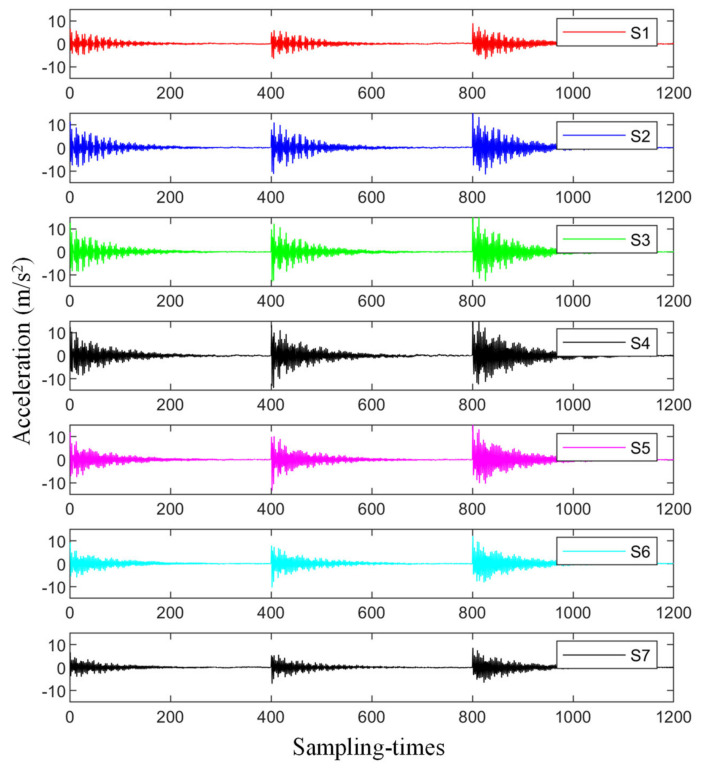
Acceleration signals from the experimental model.

Figure A3 (Appendix A) in the Appendix shows the acceleration signals of state 1 (i.e., intact structure), where S1, S2, …, S7 are the time history curves of the acquisition points (E-A, E-B, …, E-G, respectively); the complete data are shown in the Supplementary Materials. It should be noted that the magnitudes of the excitation forces of the structure were not the same for the manual excitation.

**Figure 13:**
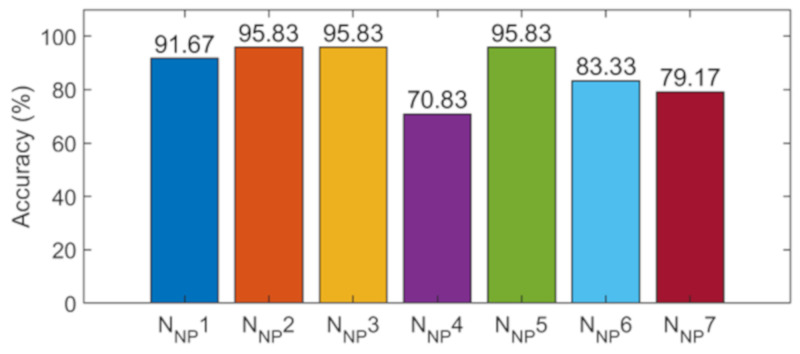
Detection results of the testing samples using 7 networks.

**Figure 14:**
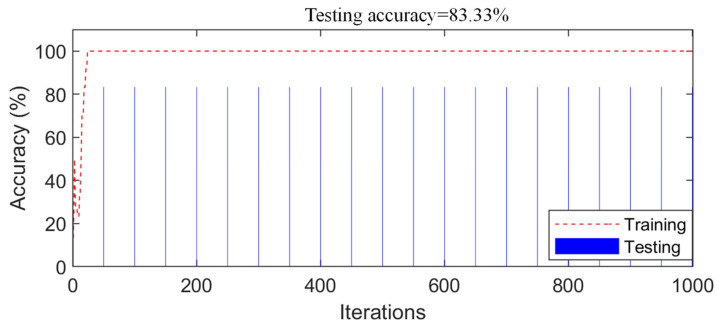
Detection results of the control experiment (experimental bridge model).

**Figure A4:**
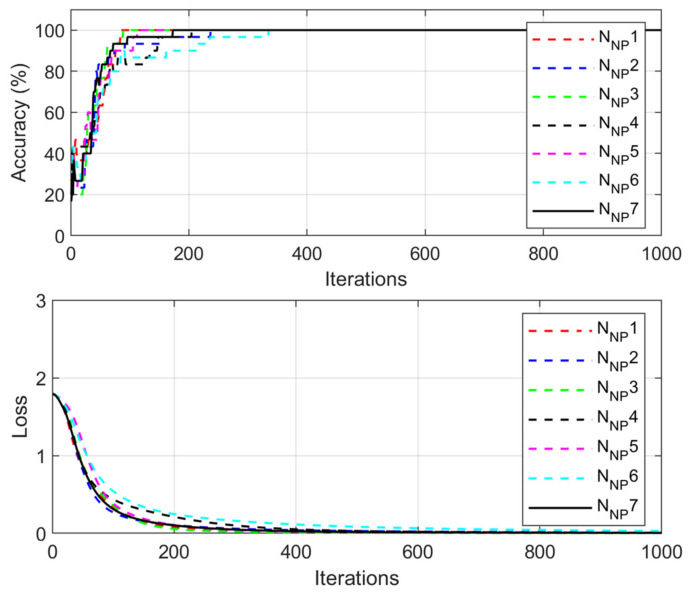
Training process and detection results of the 7 CNNs.

The training samples described in Section 2.3 were input into the seven CNN models. Figure A4 (Appendix A) shows the training process of the 1-D CNN. The accuracy increased and the loss value decreased with the increase in iterations, and finally, both tended to be stable, the accuracy reached 100%, and the loss value was close to 0. The testing samples were used to evaluate the detection performance of the networks. The detection accuracy of the testing samples is shown in Figure 13. The detection accuracy of the seven networks ranged from 70 to 96%, with the lowest of 70.83% for N_NP_4 and the highest of 95.83% for N_NP_2, N_NP_3, and N_NP_5. Then, the decision-level fusion strategy was used to fuse the prediction results of the seven networks, where the accuracy was 100%. The training process of the control experiment (data-level fusion) is shown in Figure 14; in the stable stage of network training, the accuracy of the training samples reached 100%. Figure 14 also shows the accuracy of the testing samples in the different iterations. Finally, the accuracy of the testing samples was 83.33%; therefore, the accuracy of the proposed method was higher than that of the control experiment (data-level fusion).

Table 4 shows the detection errors of the seven networks. The results show that: N_NP_1 had incorrect detections for structural states 5 and 6; N_NP_2 had an incorrect detection only for structural state 1; N_NP_4 had incorrect detections for structural states 1, 2, 4, and 5; and so on. Generally, each CNN model had different sensitivities to different damage states, and each CNN model provided the correct prediction for some specific damage states. Therefore, it was of great significance to fuse the detection results of multiple networks. The accuracy of an individual network (the detection results before fusion) was 70–96% (Table 5); the accuracy after fusion was 100% such that the accuracy was increased by 4–30% (average 12.50%). Therefore, the proposed method was validated in the experimental model.

**Figure 15:**
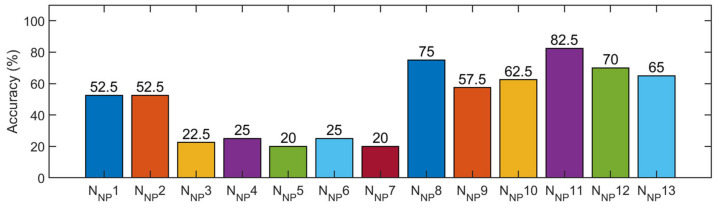
Detection results of the testing samples using the 13 networks.

**Figure A5:**
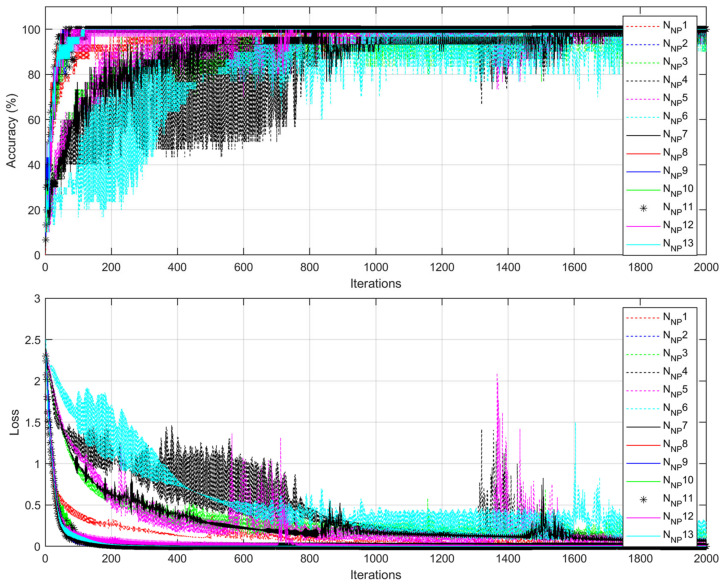
Training process and detection results.

Figure A5 (Appendix A) and Figure 15 show the training process and testing results of the steel frame model, respectively. The results showed that different CNN models had different testing accuracies, where the lowest was 20% (for N_NP_5 and N_NP_7), the highest was 82.5% (for N_NP_11), and the average was 48.46%. The networks with poor performance were N_NP_3, N_NP_4, N_NP_5, N_NP_6, and N_NP_7, and the accuracy was only 20–25%. In this study, the accuracy of the decision-level fusion strategy was improved to 85%; however, the accuracy of the control experiment (data-level fusion) was only 55%. The detailed detection results are shown in Table 6; the accuracy of the decision-level fusion strategy was higher than that of any CNN trained by the signals of an individual acquisition point (improvement of 2.5–65%, average 51.54%).

In general, the decision-level fusion of the prediction results of the multiple networks was better than the prediction results of the fusion of the original data (data-level fusion), as shown in Table 7. Compared with the data-level fusion, the proposed method improved the prediction accuracy by 10% for the numerical model, 16% for the experimental model (bridge model), and 30% for the other experimental model (steel frame model). Meanwhile, compared with the D–S evidence fusion strategy (Table 8), the proposed method improved the prediction accuracy by 1.7% for the numerical model, 0% for the experimental model (bridge model), and 75% for another experimental model (steel frame model). In particular, the D–S evidence fusion strategy was invalid for the steel frame model. The results showed that the computational efficiency (Table 9) of the proposed decision-level fusion was about 60% of the data-level fusion, while the efficiency of the D–S evidence fusion strategy was lower than that of the proposed decision-level fusion.

**Figure 16:**
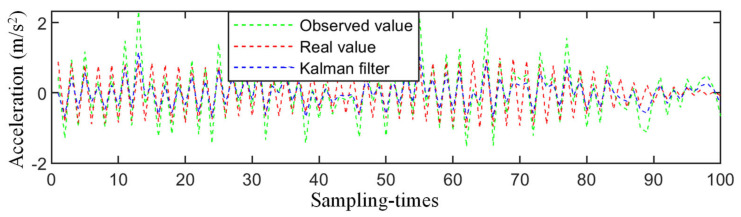
The denoise results of the Kalman filter.

The eigen perturbation and Kalman-filter-based SDD methods can process the structural vibration response signals in real time and identify the structural parameters [10,44]. Figure 16 shows the observed, real, and Kalman filter values of a vibration signal; it shows that the Kalman filter could effectively reduce the noise interference. The calculation time for each vibration signal was 2.4 s; therefore, the computational time for the three models (numerical bridge (472 samples), experimental bridge (72 samples), and steel frame (120 samples)) were 1132 s, 173 s, and 288 s, respectively. By comparison, it was found that the CNN had strong advantages in processing large-scale data (because more than 90% of the CNN’s time was spent in the training phase, once the network training was completed, its speed in the detection phase was quite fast). This confirms that this CNN has considerable advantages when used for a large amount of infrastructure monitoring data.

**Figure 17:**
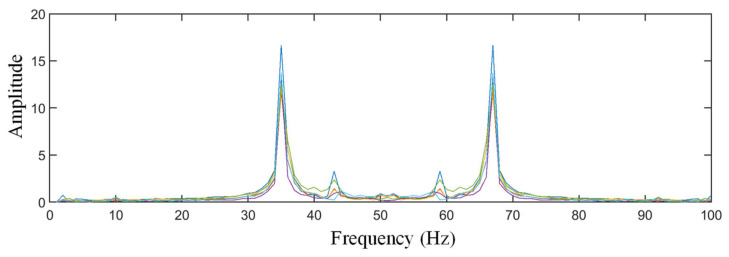
The spectrum curves of the different structural states.

Furthermore, the results of the parameter identification (Figure 17, the spectrum curves of the vibration signals of the six structural states) show that the structural damage only caused small changes in the spectrum. 

## 4. Conclusions

In this study, a 1-D CNN was employed to detect the damage of a bridge and a steel frame structure, and a novel fusion strategy (decision-level fusion) was used to fuse the prediction results of multiple CNNs, which significantly improved the accuracy of the SDD. Specifically, the vibration signal of each acquisition point was used to train a CNN, and the prediction results of these CNN models were fused.

Based on the above results, the following conclusions were drawn:

(1) The proposed fusion strategy (decision-level fusion) could significantly improve the prediction accuracy of the numerical model by 10% compared with the control experiment (data-level fusion).

(2) The proposed fusion strategy (decision-level fusion) was also validated in the experimental bridge model, and the accuracy was improved by 16% compared with the data-level fusion strategy in the control experiment. This was also confirmed regarding the damage detection of the long-span steel frame (improved by 30%).

(3) The proposed fusion strategy also performed better than any CNN trained by the signals of an individual acquisition point.

(4) The proposed method was more competitive than the D–S evidence theory and a Kalman filter. 

## Figures and Tables

**Table 1:** Structural parameters of the 1-D CNN.

Layer	Type	Kernel Num.	Kernel Size	Stride	Activation
1	Input	None	None	None	None
2	Convolution (C1)	128	3 × 1	1	Leaky ReLU
3	Max pooling (P)	None	2 × 1	1	None
4	Convolution (C2)	256	2 × 1	1	Leaky ReLU
5	FC	None	None	None	None
6	Softmax	None	None	None	None
7	Classification	None	None	None	None

**Table 2:** Cases that were mistakenly detected by the numerical model.

Sample	N_NP_1	N_NP_2	N_NP_3	N_NP_4	N_NP_5	N_NP_6	N_NP_7
1	1	1	5	1	8	5	2
2	6	4	5	1	9	6	6
3	8	6	6	5	10	9	8
4	9	7	8	5	11	11	11
5	9	16	9	10	11	11	13
6	13	18	10	13	15	14	17
7	13	18	10	15	18	15	19
8	14	19	14	20	19	17	26
9	14	19	19	21	20	18	27
10	17	22	20	21	23	20	27
11	21	23	20	22	29	23	28
12	21	25	20	23	32	25	31
13	30	25	22	24	32	25	31
14	32	26	22	31	34	27	33
15	32	31	36	31	34	28	36
16	34	35	39	34	35	31	36
17	37	36	43	34	36	32	36
18	38	39	45	36	37	37	37
19	39	40	52	42	37	40	38
20	41	45	54	43	39	49	38
21	42	46	55	44	41	49	43
22	47	49	56	48	42	50	45
23	48	51	58	53	43	52	49
24	49	54	59	54	45	54	49
25	57	55		55	46	54	50
26		55			48	57	52
27					58		53

Note: Number is the state number.

**Table 3:** Comparisons of the detection results before and after the decision-level fusion.

	Accuracy	Decision-Level Fusion	Improvement
N_NP_1	89.41%	100%	10.59%
N_NP_2	88.98%		11.02%
N_NP_3	89.83%		10.17%
N_NP_4	89.41%		10.59%
N_NP_5	88.56%		11.44%
N_NP_6	88.98%		11.02%
N_NP_7	88.56%		11.44%
Average	89.10%		10.90%

**Table 4:** Incorrect detection results of the experimental model.

Sample	N_NP_1	N_NP_2	N_NP_3	N_NP_4	N_NP_5	N_NP_6	N_NP_7
1	6	1	4	1	4	1	1
2	5			4		3	3
3				1		5	5
4				2		6	5
5				5			5
6				1			
7				1			

**Table 5:** Comparisons of the detection results before and after the decision-level fusion.

	Accuracy	Decision-Level Fusion	Improved
N_NP_1	91.67%	100%	8.33%
N_NP_2	95.83%		4.17%
N_NP_3	95.83%		4.17%
N_NP_4	70.83%		29.17%
N_NP_5	95.83%		4.17%
N_NP_6	83.33%		16.67%
N_NP_7	79.17%		20.83%
Average	87.50%		12.50%

**Table 6:** Comparisons of the detection results before and after the decision-level fusion.

	Accuracy	Decision-Level Fusion	Improvement
N_NP_1	52.50%	85%	47.50%
N_NP_2	52.50%		47.50%
N_NP_3	22.50%		77.50%
N_NP_4	25.00%		75.00%
N_NP_5	20.00%		80.00%
N_NP_6	25.00%		75.00%
N_NP_7	20.00%		80.00%
N_NP_8	75.00%		25.00%
N_NP_9	57.50%		42.50%
N_NP_10	62.50%		37.50%
N_NP_11	82.50%		17.50%
N_NP_12	70.00%		30.00%
N_NP_13	65.00%		35.00%
Average	48.46%		51.54%

**Table 7:** Comparisons of the accuracy with the control experiment (data-level fusion).

	Numerical Model (Bridge Model)	Experimental Model (Bridge Model)	Experimental Model (Steel Frame)
Data-level fusion	89.83%	83.33%	55.00%
Decision-level fusion	100.00%	100.00%	85.00%
Improvement	10%	16%	30%

**Table 8:** Comparisons of the accuracy with the D–S evidence fusion.

	Numerical Model (Bridge Model)	Experimental Model (Bridge Model)	Experimental Model (Steel Frame)
Decision-level fusion	100.00%	100.00%	85.00%
D–S evidence fusion	98.31%	100.00%	10.00%
Improvement	1.69%	0%	75%

**Table 9:** Comparisons of the computational efficiency with the three fusion strategies.

	Numerical Model (Bridge Model)	Experimental Model (Bridge Model)	Experimental Model (Steel Frame)
Decision-level fusion	721 s	350 s	1300 s
Data-level fusion	488 s	240 s	510 s
D–S evidence fusion	1073 s	369 s	1362 s

## Data Availability

Some or all data, models, or codes generated or used during the study are available from the corresponding author by request.

## Supplementary Materials

The following are available online at https://www.mdpi.com/article/10.3390/s21123950/s1.

## References

[1] Yan Y.J., Cheng L., Wu Z.Y., Yam L.H. (2007). Development in vibration-based structural damage detection technique. Mech. Syst. Signal Process..

[2] An Y., Chatzi E., Sim S.-H., Laflamme S., Blachowski B., Ou J. (2019). Recent progress and future trends on damage identification methods for bridge structures. Struct. Control Health Monit..

[3] Pandey A.K., Biswas M., Samman M.M. (1991). Damage detection from changes in curvature mode shapes. J. Sound Vib..

[4] Sung S.H., Koo K.Y., Jung H.J. (2014). Modal flexibility-based damage detection of cantilever beam-type structures using baseline modification. J. Sound Vib..

[5] Lu Q., Ren G., Zhao Y. (2002). Multiple Damage Location with Flexibility Curvature and Relative Frequency Change for Beam Structures. J. Sound Vib..

[6] Teng S., Chen G., Liu G., Lv J., Cui F. (2019). Modal Strain Energy-Based Structural Damage Detection Using Convolutional Neural Networks. Appl. Sci..

[7] Cha Y., Buyukozturk O. (2015). Structural Damage Detection Using Modal Strain Energy and Hybrid Multiobjective Optimization. Comput. Aided Civ. Infrastruct. Eng..

[8] Ni F., Zhang J., Noori M.N. (2020). Deep learning for data anomaly detection and data compression of a long-span suspension bridge. Comput. Aided Civ. Infrastruct. Eng..

[9] Khuc T., Catbas N. (2017). Completely contactless structural health monitoring of real-life structures using cameras and computer vision. Struct. Control Health Monit..

[10] Feng G., Yong L. (2006). A Kalman-filter based time-domain analysis for structural damage diagnosis with noisy signals. J. Sound Vib..

[11] Ghiasi R., Torkzadeh P., Noori M. (2016). A machine-learning approach for structural damage detection using least square support vector machine based on a new combinational kernel function. Struct. Health Monit..

[12] Yam L.H., Yan Y.J., Jiang J.S. (2003). Vibration-based damage detection for composite structures using wavelet transform and neural network identification. Compos. Struct..

[13] Mehrjoo M., Khaji N., Moharrami H., Bahreininejad A. (2008). Damage detection of truss bridge joints using Artificial Neural Networks. Expert Syst. Appl..

[14] Gonzalez M.P., Zapico J.L. (2008). Seismic damage identification in buildings using neural networks and modal data. Comput. Struct..

[15] Chun P.J., Yamashita H., Furukawa S. (2015). Bridge Damage Severity Quantification Using Multipoint Acceleration Measurement and Artificial Neural Networks. Shock Vib..

[16] Lautour O., Omenzetter P. (2010). Damage classification and estimation in experimental structures using time series analysis and pattern recognition. Mech. Syst. Signal Process..

[17] Katunin A., Araújo dos Santos J.V., Lopes H. (2021). Damage identification by wavelet analysis of modal rotation differences. Structures.

[18] Dackermann U., Li J., Samali B. (2010). Dynamic-Based Damage Identification Using Neural Network Ensembles and Damage Index Method. Adv. Struct. Eng..

[19] Zhong K., Teng S., Liu G., Chen G., Cui F. (2020). Structural Damage Features Extracted by Convolutional Neural Networks from Mode Shapes. Appl. Sci..

[20] Lin Y.Z., Nie Z.H., Ma H.W. (2017). Structural Damage Detection with Automatic Feature extraction through Deep Learning. Comput. Aided Civ. Infrastruct. Eng..

[21] Teng S., Liu Z., Chen G., Cheng L. (2021). Concrete Crack Detection Based on Well-Known Feature Extractor Model and the YOLO_v2 Network. Appl. Sci..

[22] Yi M.W., Samali B. (2002). Shake table testing of a base isolated model. Eng. Struct..

[23] Yu Y., Wang C., Gu X., Li J. (2019). A novel deep learning-based method for damage identification of smart building structures. Struct. Health Monit..

[24] Abdeljaber O., Sassi S., Avci O., Kiranyaz S., Ibrahim A.A., Gabbouj M. (2019). Fault Detection and Severity Identification of Ball Bearings by Online Condition Monitoring. IEEE Trans. Ind. Electron..

[25] Kiranyaz S., Gastli A., Ben-Brahim L., Alemadi N., Gabbouj M. (2018). Real-Time Fault Detection and Identification for MMC using 1D Convolutional Neural Networks. IEEE Trans. Ind. Electron..

[26] Abdeljaber O., Avci O., Kiranyaz S., Gabbouj M., Inman D.J. (2017). Real-time vibration-based structural damage detection using one-dimensional convolutional neural networks. J. Sound Vib..

[27] Avci O., Abdeljaber O., Kiranyaz S., Hussein M., Inman D.J. (2018). Wireless and real-time structural damage detection: A novel decentralized method for wireless sensor networks. J. Sound Vib..

[28] Zhang Y., Miyamori Y., Mikami S., Saito T. (2019). Vibration-based structural state identification by a 1-dimensional convolutional neural network. Comput. Aided Civ. Infrastruct. Eng..

[29] Nemec S.F., Donat M.A., Mehrain S., Friedrich K., Krestan C., Matula C., Imhof H., Czerny C. (2007). CT-MR image data fusion for computer assisted navigated neurosurgery of temporal bone tumors. Eur. J. Radiol..

[30] Ashraf S., Brabyn L., Hicks B.J. (2011). Image data fusion for the remote sensing of freshwater environments. Appl. Geogr..

[31] Tang Z., Chen Z., Bao Y., Li H. (2019). Convolutional neural network-based data anomaly detection method using multiple information for structural health monitoring. Struct. Control Health Monit..

[32] Ernesto G., Maura I. (2014). A multi-stage data-fusion procedure for damage detection of linear systems based on modal strain energy. J. Civil Struct. Health Monit..

[33] Teng S., Chen G., Gong P., Liu G., Cui F. (2019). Structural damage detection using convolutional neural networks combining strain energy and dynamic response. Meccanica.

[34] Huo Z., Zhang Y., Shu L. Bearing Fault Diagnosis using Multi-sensor Fusion based on weighted D-S Evidence Theory. Proceedings of the 2018 18th International Conference on Mechatronics-Mechatronika (ME).

[35] Li J., Zhu X., Law S.S. (2020). A two-step drive-by bridge damage detection using Dual Kalman Filter. Int. J. Struct. Stab. Dyn..

[36] Ying L., Feng C., Zhou H. (2015). An algorithm based on two-step Kalman filter for intelligent structural damage detection. Struct. Control Health Monit..

[37] Xing S.T., Marvin W. (2013). Application of substructural damage identification using adaptive Kalman filter. J. Civ. Struct. Health Monit..

[38] Sen S., Bhattacharya B. (2017). Online structural damage identification technique using constrained dual extended Kalman filter. Struct. Control Health Monit..

[39] Lai Z., Lei Y., Zhu S. (2016). Moving-window extended Kalman filter for structural damage detection with unknown process and measurement noises. Measurement.

[40] Al-Hussein A., Haldar A. (2015). Novel Unscented Kalman Filter for Health Assessment of Structural Systems with Unknown Input. J. Eng. Mech..

[41] Huo Z., Miguel M.G., Zhang Y. (2020). Entropy Measures in Machine Fault Diagnosis: Insights and Applications. IEEE Trans. Instrum. Meas..

[42] Nie Z., Ngo T., Ma H. (2017). Reconstructed Phase Space-Based Damage Detection Using a Single Sensor for Beam-Like Structure Subjected to a Moving Mass. Shock Vib..

[43] Scherer D., Müller A., Behnke S. Evaluation of Pooling Operations in Convolutional Architectures for Object Recognition. Proceedings of the International Conference on Artificial Neural Networks.

[44] Huang J., Li D., Zhang C. (2019). Improved Kalman filter damage detection approach based on *l_p_* regularization. Struct. Control Health Monit..

